# Cellular and circuit diversity determines the impact of endogenous opioids in the descending pain modulatory pathway

**DOI:** 10.3389/fnsys.2022.963812

**Published:** 2022-08-15

**Authors:** Kylie B. McPherson, Susan L. Ingram

**Affiliations:** ^1^Division of Neuroscience and Clinical Pharmacology, Department of Biomedical Sciences, University of Cagliari, Monserrato, Italy; ^2^Department of Neurological Surgery, Oregon Health & Science University, Portland, OR, United States

**Keywords:** vlPAG, cellular diversity, circuit diversity, endogenous opioids, descending pain modulation

## Abstract

The descending pain modulatory pathway exerts important bidirectional control of nociceptive inputs to dampen and/or facilitate the perception of pain. The ventrolateral periaqueductal gray (vlPAG) integrates inputs from many regions associated with the processing of nociceptive, cognitive, and affective components of pain perception, and is a key brain area for opioid action. Opioid receptors are expressed on a subset of vlPAG neurons, as well as on both GABAergic and glutamatergic presynaptic terminals that impinge on vlPAG neurons. Microinjection of opioids into the vlPAG produces analgesia and microinjection of the opioid receptor antagonist naloxone blocks stimulation-mediated analgesia, highlighting the role of endogenous opioid release within this region in the modulation of nociception. Endogenous opioid effects within the vlPAG are complex and likely dependent on specific neuronal circuits activated by acute and chronic pain stimuli. This review is focused on the cellular heterogeneity within vlPAG circuits and highlights gaps in our understanding of endogenous opioid regulation of the descending pain modulatory circuits.

## Descending Pain Modulation

Noxious stimuli evoke a sensory experience perceived as pain. Noxious signals initiated in the periphery are transmitted to many supraspinal structures that process the sensory, cognitive, affective, and motivational components that concurrently shape pain perception. These higher-order brain regions collectively project to the descending pain modulatory pathway, consisting of the ventrolateral column of the periaqueductal grey (vlPAG; Bandler et al., 1991; Bandler and Shipley, 1994) and the rostroventromedial medulla (RVM). RVM efferents to the dorsal horn of the spinal cord facilitate or inhibit incoming nociceptive inputs from the periphery (Basbaum and Fields, 1979; Mantyh and Peschanski, 1982; Vanegas et al., 1984b). The net output of this circuit under various acute and chronic pain conditions has been well-studied, especially the role of the RVM in bidirectional pain modulation (Basbaum and Fields, 1984; Lau and Vaughan, 2014; Heinricher and Ingram, 2020).

Bidirectional pain modulation by the RVM has been demonstrated through many studies using stimulation, pharmacology, *in vivo* electrophysiology, and various pain models. Neurons in the RVM have been characterized with respect to their role in modulating pain, identifying three distinct types of neurons: ON-, OFF-, and NEUTRAL-cells. These neuron types were initially characterized by their unique responses (*in vivo*) to acute noxious stimuli (Fields et al., 1983a; Vanegas et al., 1984b). ON-cells increase firing just prior to the tail-flick or paw withdrawal behavioral response to an acute noxious stimulus, OFF-cells pause just before the ON-cell burst and withdrawal response, and NEUTRAL-cells show no changes in firing. Some of each cell type project down to the dorsal horn (Vanegas et al., 1984b; Fields et al., 1995). Increased OFF-cell firing suppresses nociceptive reflexes (Fields and Heinricher, 1985) producing *descending inhibition of pain*, which can be seen most clearly in response to morphine or other opioids. Conversely, ON-cell firing contributes to *descending facilitation of pain*, which can be produced by many different pain models (Morgan and Fields, 1994; Porreca et al., 2002; Edelmayer et al., 2009) or pharmacological manipulations. Further, hyperalgesia and allodynia can result from either a reduction in OFF-cell firing or enhanced ON-cell firing (Martenson et al., 2009; Cleary and Heinricher, 2013). Importantly, although hyperexcited ON-cells promote descending facilitation, increased OFF-cell activation overrides the facilitation, yielding a net inhibitory output for the descending circuit (Satoh et al., 1983; Hentall et al., 1984; Fields and Heinricher, 1985; Jensen and Yaksh, 1989; Heinricher and Ingram, 2020).

In contrast to the bidirectional modulation of pain by the RVM, the upstream vlPAG has been primarily implicated in producing descending inhibition, due to the analgesic effect of both vlPAG stimulation (electrical or chemical) and locally applied opioid agonists (Reynolds, 1969; Mayer et al., 1971; Mayer and Liebeskind, 1974; Akil and Liebeskind, 1975; Soper and Melzack, 1982; Vanegas et al., 1984a; Jensen and Yaksh, 1989; Bandler et al., 1991; Bandler and Shipley, 1994; Tortorici and Morgan, 2002). vlPAG stimulation-mediated analgesia occurs predominantly *via* the dense projection to the RVM (Behbehani and Fields, 1979; Gebhart et al., 1983; Prieto et al., 1983), as the vlPAG sends sparse efferents directly to the dorsal horn (Basbaum and Fields, 1979). In particular, vlPAG stimulation activates an excitatory connection between the vlPAG and RVM OFF-cells (Behbehani and Fields, 1979; Basbaum and Fields, 1984; Vanegas et al., 1984a). More recent studies have reinforced the antinociceptive role of vlPAG glutamate neurons using selective, chemogenetic activation and have demonstrated that selective activation of vlPAG GABAergic neurons can produce hyperalgesia (Samineni et al., 2017a)—suggesting the capacity for the vlPAG to be an additional locus of bidirectional pain modulation. Interestingly in opposition to the role vlPAG glutamate neurons play in descending inhibition, a recent study has identified a subpopulation of dynorphin-expressing v/lPAG glutamate neurons, which when chemogenetically activated facilitate nociception (Nguyen et al., 2022). The key question remains whether distinct vlPAG populations, such as RVM-projecting glutamatergic or GABAergic neurons or more specific subpopulations within these groups, are activated by acute noxious stimuli or in persistent and chronic pain conditions mirroring these different experimental manipulations that produce descending inhibition or facilitation.

The vlPAG is a key site of opioid-induced analgesia mediated by mu-opioid receptors (MOR; Heinricher and Morgan, 1999). Activation of postsynaptic MORs, expressed on a subpopulation of vlPAG neurons, produces a hyperpolarizing current; whereas, activation of MORs in presynaptic terminals within the vlPAG inhibits neurotransmitter release. Pre- and postsynaptic MORs coupled to different signaling pathways work in concert to promote descending inhibition. However, other forms of global inhibition of the vlPAG (e.g., muscimol or baclofen) result in descending facilitation of pain, not the inhibition (analgesia) produced by opioid infusion. These contradictory findings were explained by a circuit mechanism referred to as the opioid-mediated *disinhibition of pain hypothesis* (Basbaum and Fields, 1984). This mechanism hypothesizes that opioids inhibit GABA release onto vlPAG neurons, either through selective postsynaptic MOR expression on inhibitory interneurons or at the level of the presynaptic GABAergic afferent terminals, disinhibiting excitatory RVM-projecting neurons that promote descending inhibition of pain. In subsequent sections, we consider critical studies that identify additional complexity in MOR expression, signaling, and regulation, that provide many loci for pain-mediated alterations to influence opioid-mediated pain modulation.

The descending pain modulatory circuit also exhibits sexual dimorphism (Fullerton et al., 2018). Females have ~33% more RVM-projecting neurons than males, however, persistent inflammation activates significantly more RVM-projecting neurons in males than females (Loyd and Murphy, 2006, 2014). In addition, the antinociceptive potency of bicuculline, a GABA_A_ receptor antagonist, injected into the vlPAG is greater in male rats compared to females (Bobeck et al., 2009), indicating differences in GABA tone within the vlPAG between males and females (Tonsfeldt et al., 2016). This same study showed that the antinociceptive potency of kainic acid, which activates glutamate receptors, is the same for males and females. This indicates that direct activation of the vlPAG activates a sufficient number of RVM OFF-cells needed to suppress nociception, which has been estimated to require relatively few neurons (<100; Hentall et al., 1984). Furthermore, RVM-projecting vlPAG neurons are more strongly disinhibited by systemic morphine in male rats compared to females (Loyd et al., 2007), emphasizing the need to continue to improve our understanding of how descending modulation of pain varies between males and females.

To date, studies have focused on defining the net output of the vlPAG and RVM in response to nociceptive stimuli and different pain states. However, many features of cellular- and circuit-based diversity suggest additional layers of complexity relevant to increasing our understanding of the role of the vlPAG in descending pain modulation in uninjured and pain states. Specifically, features including cortical and subcortical afferent inputs, efferent targets, neurotransmitter content, receptor and channel expression, morphology, intrinsic membrane properties, and responses to stimuli, can be used to discern between subpopulations of vlPAG neurons (Hamilton, 1973; Heinricher et al., 1987; Barbaresi and Manfrini, 1988; Chieng and Christie, 1994a; Park et al., 2010; Heinricher and Ingram, 2020; McPherson et al., 2021). The vlPAG is involved in many behavioral circuits associated with survival (i.e., threat, fear, pain) as well as vital autonomic functions like breathing, feeding, and respiration (Bandler et al., 2000; George et al., 2019; Silva and McNaughton, 2019). Thus, it is a prime brain area for using genetically encoded circuit-mapping tools to understand how specific PAG afferents participate in different behaviors. The broad categorization of excitatory and inhibitory neurons defined by these genetic methods is a useful starting point, however, does not account for the diversity that further distinguishes subpopulations of vlPAG neurons. In addition, methods dependent on gene expression (such as neurotransmitter content or MOR expression) assume that large populations defined by one key descriptive feature activate or inactivate in unison in response to stimuli, whereas studies are increasingly showing that this interpretation does not hold (Vaaga et al., 2020; McPherson et al., 2021). This review is focused on what is known about different aspects of heterogeneity within the vlPAG in relation to the descending pain modulatory system.

## Opioids in The Descending Pain Modulatory Pathway

### Endogenous opioids

Stimulation of the vlPAG produces analgesia in humans and antinociception in rats that is blocked by the MOR antagonist naloxone (Adams, 1976; Akil et al., 1976; Hosobuchi et al., 1977; Behbehani and Fields, 1979; Barbaro, 1988; Bach and Yaksh, 1995), providing evidence for the release of endogenous opioids in the vlPAG (Bagley and Ingram, 2020). Stimulation of the PAG produces an increase in the release of met-enkephalin (ME; Bach and Yaksh, 1995), a full MOR agonist, that is typically below detection limits under basal conditions (Del Rio et al., 1983). It is not clear where the ME originates as a subset of neurons distributed throughout the vlPAG express enkephalin and enkephalin-containing afferent terminals from other brain areas (Moss et al., 1983; Williams and Dockray, 1983). Enkephalin-containing vlPAG neurons send projections to the amygdala and the nucleus accumbens (Li et al., 1990a, b) but they may also send local collaterals. Both of these areas send inputs to vlPAG indicating multiple reciprocal circuits exist between the vlPAG and supraspinal brain areas where endogenous opioids may influence descending modulation. This is further supported by studies showing activation of enkephalin-expressing inhibitory interneurons in the central nucleus of the amygdala (CeA) increases Fos expression in non-serotonergic vlPAG neurons, inducing analgesia (Poulin et al., 2008; Paretkar and Dimitrov, 2019).

The vlPAG also receives β-endorphin-containing fibers from the arcuate nucleus of the hypothalamus (Finley et al., 1981; Ibata et al., 1985; Sim and Joseph, 1991), with confirmed β-endorphin release in the vlPAG following stimulation of the arcuate nucleus (Bach and Yaksh, 1995). The hypothalamus sends endomorphin-2 containing projections to the PAG (Chen et al., 2008) and high levels of endomorphin-2 are observed within the PAG (Martin-Schild et al., 1999). β-endorphin is a full agonist while endomorphin-2 is a partial agonist of the MOR (Narita et al., 2000) suggesting that these two agonists will activate MORs differently. In addition to endogenous MOR agonists, electrical stimulation of the CeA increases levels of the kappa-opioid receptor (KOR) agonist dynorphin A in the lateral PAG (Nakamura et al., 2013), however, dynorphin microinjection within the PAG is not analgesic (Fang et al., 1989). A recent study discovered a subpopulation of dynorphin-expressing vlPAG glutamate neurons that can facilitate nociception through KOR signaling within the RVM (Nguyen et al., 2022).

As one would anticipate, the release of endogenous opioid peptides in response to pain states and exogenous opioids varies significantly. Substance P induces ME release in the PAG that is correlated with antinociception (Del Rio et al., 1983; Rosén et al., 2004). The CFA-induced inflammatory pain model increases neuropeptide release (neurotensin increased 133% and ME increased 353%), with differential time courses for recovery (Williams et al., 1995). After 7 d of inflammation, neurotensin returns to baseline but ME remains elevated. Further, the enhancement in ME release in the vlPAG with inflammation is seen uniformly across the rostral-caudal axis and is maintained between 4 h, 4 d, and 14 d post-CFA in male rats (Hurley and Hammond, 2001). Other opioid peptides are also increased with other pain models. Formalin-induced inflammation increases the release of β-endorphin and endomorphin-2 within the PAG (Sun et al., 2001; Nakamura et al., 2013) and β-endorphin is released within the vlPAG during stress-induced analgesia (Külling et al., 1989). Interestingly, endogenous ME release is increased ~50% by systemic morphine injection (Williams et al., 1995). Similarly, antinociception produced by DAMGO injections into the basolateral amygdala (BLA) is occluded by blocking endogenous MOR activation with MOR antagonists in the vlPAG (Tershner and Helmstetter, 2000), demonstrating synergy between exogenous and endogenous opioid effects. The extent of the endogenous opioid release and efficacy during naïve and pain states with and without exogenous opioid use are key points of an ongoing investigation.

Importantly, extensive work is still required to understand endogenous opioid peptides in descending modulation in females, as most of the early studies were conducted in male rats. This is of particular importance given the sex differences observed in pain states both in animal models and the clinical population (Fullerton et al., 2018; Shansky and Murphy, 2021). Similarly, these studies are largely carried out in adult rats, overlooking the possible differences in the development of the descending pain modulatory circuit. One prime example of this is that activation of opioid receptors in the PAG produces opposite effects in young rats compared to adults (Kwok et al., 2014). This is a significant area of research considering 37% of children in the clinical population experience chronic pain (King et al., 2011). Our understanding of the mechanisms by which endogenous opioids produce analgesia during pain states is further complicated by the role of endogenous opioids in other circumstances (i.e., stress-induced analgesia; Ferdousi and Finn, 2018).

Endogenous opioids must be released with the spatial and temporal precision necessary to activate the circuit without directly inhibiting excitatory PAG efferents that target RVM OFF-cells involved in descending pain inhibition. Furthermore, how the release and efficacy of endogenous opioids are impacted by acute or ongoing pain states is not understood. Complementary to release, it is crucial to understand the specificity of opioid receptor expression and signaling across diverse neuron populations and cellular compartments with distinct mechanisms of action. Next, we consider many important studies that shape our understanding of MOR action in the vlPAG.

### MOR expression and signaling

The PAG contains a high density of MOR expressing neurons (Mansour et al., 1986; Kalyuzhny et al., 1996; Gutstein et al., 1998; Commons et al., 1999, 2000; Wang and Wessendorf, 2002). As previously discussed, the disinhibition of pain hypothesis provides a possible circuit mechanism for opioid-mediated analgesia at the level of the vlPAG. Specifically, this mechanism proposes selective MOR expression on the cell bodies of vlPAG inhibitory interneurons within the vlPAG (Basbaum and Fields, 1984) and was later updated to include expression on GABAergic presynaptic terminals within the vlPAG (Chieng and Christie, 1994b; Lau and Vaughan, 2014).

MORs are G_i/o_-coupled G protein-coupled receptors (GPCRs) expressed on postsynaptic cell bodies within the vlPAG. Agonist-bound MORs initiate a cascade of many signal transduction processes, including the activation of G protein-coupled inwardly-rectifying potassium channels (GIRKs) by βγ-subunits of activated G_i/o_ G proteins (Logothetis et al., 1987) that produces a K^+^ efflux and subsequent hyperpolarization of the neuron, inhibiting firing (North et al., 1987). MOR agonists exhibit functional selectivity differences in G_i/o_ recruitment in MOR-GIRK signaling. In particular, maximal GIRK currents induced by DAMGO and fentanyl require G_o_ G proteins, compared to ME, which requires G_i_ (McPherson et al., 2018).

MORs are also expressed on presynaptic terminals within the vlPAG where they inhibit the release of the neurotransmitter (Chieng and Christie, 1994b; Vaughan et al., 1997). In GABAergic terminals, MORs couple to voltage-gated potassium channels through the phospholipase A_2_→ arachidonic acid → 12-lipoxygenase cascade (Vaughan et al., 1997). This signaling pathway is not necessary for MOR inhibition of glutamate release in the vlPAG and is distinct from that used by other presynaptic GPCRs that inhibit GABA release (i.e., GABA_B_; Vaughan et al., 1997; Bouchet and Ingram, 2020). Agonist-specific functional selectivity in the recruitment of G_i_ or G_o_ G proteins also occurs during the inhibition of presynaptic GABA release. Specifically, in order to achieve maximal efficacy for inhibiting spontaneous GABA release DAMGO requires G_o_, fentanyl requires both G_o_ and G_i_, and ME sufficiently inhibits release with either (Bouchet et al., 2021). Comparatively, for maximal inhibition of evoked GABA release, DAMGO requires both G_o_ and G_i_, and fentanyl and ME require G_i_.

Postsynaptic MOR expression has been found on ~30%–60% of vlPAG neurons where MOR agonist application produces a GIRK current response reversible by a MOR antagonist (Chieng and Christie, 1994a; McPherson et al., 2018). Many studies have concluded that this subset of MOR-expressing neurons is GABAergic interneurons that tonically inhibit glutamatergic projection neurons (Yaksh et al., 1976; Basbaum and Fields, 1984; Reichling et al., 1988; Park et al., 2010; Lau and Vaughan, 2014). This selective neuron-type expression has been challenged by several studies showing evidence of postsynaptic MORs on neurons with varied neurotransmitter content, intrinsic firing properties, and morphology (Chieng and Christie, 1994a; Osborne et al., 1996; Commons et al., 2000; Morgan et al., 2008; Zhang et al., 2020; McPherson et al., 2021). Furthermore, MOR activation has been shown to directly inhibit a subset (~14%) of RVM-projecting vlPAG neurons (Osborne et al., 1996). However, this does not rule out the possibility that MORs can be expressed on GABAergic projection neurons that send local collaterals within the vlPAG. Thus, it is clear that the actions of opioids in vlPAG are more complex than their ability to disinhibit excitatory RVM-projecting vlPAG neurons.

The analgesic effect of morphine microinjected into the PAG is reversed by muscimol also microinjected into the PAG (Moreau and Fields, 1986), underscoring the overall role of opioids in alleviating inhibitory tone to produce analgesia. As a result of both pre- and postsynaptic mechanisms, MOR activation disinhibits excitatory RVM-projecting vlPAG neurons (Lau et al., 2020), which can activate downstream nociception-inhibiting OFF-cells within the RVM (Fields et al., 1983b; Basbaum and Fields, 1984; Cheng et al., 1986). The non-selective excitatory amino acid (EAA) receptor antagonist kynurenate in the RVM abolishes systemic opioid-mediated activation of OFF-cells and antinociception (Heinricher et al., 1999), confirming the antinociceptive role of glutamate release from afferents within the RVM. However, both GABAergic and non-GABAergic vlPAG neurons have also been found to project to ON- and OFF-cells within the RVM, with varied MOR expression on their cell bodies and axon terminals within the RVM (Commons et al., 2000; Zhang et al., 2020). Parallel descending circuits, both excitatory and inhibitory vlPAG afferents within the RVM, have been discussed to encompass these findings (Lau and Vaughan, 2014).

Although MORs are most effective in inhibiting presynaptic GABA release in the vlPAG, they also inhibit release to a lesser extent from glutamatergic afferents (Lau et al., 2020). This suggests that in the presence of opioids, there is a net excitatory effect (increased E/I balance). Additionally, the EC_50_ for DAMGO-mediated inhibition of presynaptic release is roughly four times lower than that for postsynaptic K^+^ current (Pennock and Hentges, 2011). This creates the possibility for a MOR-expressing neuron to be either disinhibited by a low dose of opioids (removing inhibitory afferent tone) or inhibited by a higher dose (triggering a hyperpolarizing GIRK-mediated K^+^ current). Interestingly, there is functional selectivity between opioid agonists for pre- vs. postsynaptic signaling. To achieve maximal antinociceptive efficacy morphine requires presynaptic MOR activation and fentanyl requires postsynaptic MOR activation (Morgan et al., 2020). Overall, the activation of presynaptic MORs alone sufficiently produces analgesia. These findings present interesting questions about how smaller concentrations of targeted endogenous opioid release may alter vlPAG neuron activity differently than larger concentrations of globally delivered exogenous opioids. These compartment-specific differences in opioid potency also demonstrate the ability for opioids to have many different effects on vlPAG neurons and the subsequent signaling they trigger at efferent targets depending on E/I balance and postsynaptic MOR expression.

Interestingly, KOR, and not DOR, activation inhibits evoked inhibitory synaptic release from afferent terminals comparably to MOR activation (Lau et al., 2020). Despite this overlap in presynaptic function, vlPAG KOR activation does not produce analgesia in rats (Bodnar et al., 1988; Smith et al., 1988; Fang et al., 1989; Ossipov et al., 1995). Optogenetic studies examining KOR and MOR sensitivity of specific afferents may be able to solve this contradictory observation. It is likely that KORs are expressed on different afferent terminals from brain areas that do not have a strong role in opioid analgesia, further reinforcing the importance of identifying whether inhibiting selective vlPAG afferent inputs are necessary to produce analgesia, how these inputs are altered by pain states, and whether these alterations impact the ability for endogenous opioids to sufficiently dampen their signal.

Pain-state-mediated alterations to these parallel circuits, such as the E/I balance onto RVM-projecting vlPAG neurons or vlPAG afferent inputs onto specific neuron types in the RVM, have yet to be defined but seem likely due to known changes in opioid efficacy in these regions during pain states. Persistent inflammation (24 h) prior to systemic morphine administration significantly increases the analgesic response compared to uninjured animals (Eidson and Murphy, 2013). A study completed in male rats showed greater analgesic efficacy by DAMGO locally infused downstream in the RVM 14 d after CFA-induced inflammatory pain (Hurley and Hammond, 2001). The attenuation of morphine tolerance by persistent peripheral inflammation aligns with clinical literature, where chronic pain patients do not readily demonstrate opioid tolerance (Collett, 1998; Dworkin et al., 2005). Altogether, the effect of opioids within the vlPAG is much more complex than selective postsynaptic MOR expression inhibiting GABA interneurons. The next critical questions include whether the E/I balance is distributed uniquely across distinct subpopulations of vlPAG neurons, how opioid modulation of neuronal activity is impacted by alterations induced by pain states (i.e., altered intrinsic activity neuronal activity or presynaptic inputs), and how this influences vlPAG efferent engagement with functionally distinct ON- and OFF-cells within the RVM. In the next section, we consider different MOR signaling regulation mechanisms that reveal additional compartmental specificity in MOR signaling.

### Regulation of MOR signaling

Multiple mechanisms exist to regulate ongoing MOR signaling. Continuous MOR activation triggers the phosphorylation of the intracellular C-terminal tail of the receptor by several different protein kinases, including protein kinase A, protein kinase C (PKC), and G protein receptor kinases (Williams et al., 2013). Phosphorylation of the C-terminus triggers desensitization and recruitment of β-arrestin (βarr), resulting in the internalization of the receptor. MOR signaling is recovered around 60 m following maximal desensitization, indicating the time course for receptor recycling back to the membrane. Postsynaptic MOR-mediated GIRK currents within the vlPAG are relatively small, however, they do desensitize during prolonged MOR agonist exposure, and this desensitization is even greater in morphine-tolerant rats (Ingram et al., 2008). Desensitized MOR-GIRK signaling, enhanced by morphine tolerance, reduces the ability for opioids to hyperpolarize vlPAG neurons, suppressing their firing rates.

Although postsynaptic MORs in the vlPAG desensitize, the inhibition of GABA release by presynaptic MORs within the vlPAG does not desensitize during prolonged exposure to an agonist in drug naïve or chronic morphine treated rats (Fyfe et al., 2010). Additional evidence from the arcuate nucleus of the hypothalamus confirms presynaptic MORs, as well as other presynaptic GPCRs, are resistant to desensitization (Pennock et al., 2012). Interestingly, presynaptic MORs have been shown to undergo internalization in the continued presence of ligand, but are quickly replaced by lateral diffusion along the axon surface (Jullié et al., 2020). Thus, despite the dynamic movement of MORs in the presynaptic compartment, signaling is maintained.

In addition to βarr-mediated desensitization, activated G proteins that bind and activate effector targets are also regulated by the regulator of G protein signaling (RGS) proteins. RGS proteins bind to active α-subunits driving GTP-hydrolysis to GDP, boosting the affinity between the α- and βγ-subunits resulting in the reformation of the inactive heterotrimer. Many RGS proteins are involved in the regulation of MORs, including RGS4 (Garzón et al., 2005a; Roman et al., 2007; Leontiadis et al., 2009; Santhappan et al., 2015), RGS9-2 (Psifogeorgou et al., 2007; Papachatzaki et al., 2011; Gaspari et al., 2017), RGS19 (Wang and Traynor, 2013), and RGSz (Garzón et al., 2005b; Gaspari et al., 2018; Sakloth et al., 2019). Within the vlPAG, a mouse model with RGS-insensitive G proteins exhibits increased opioid-mediated inhibition of presynaptic GABA release and increased morphine antinociception (Lamberts et al., 2011). These findings support the idea that RGS proteins negatively modulate MOR inhibition of evoked GABA release (eIPSCs), influencing supraspinal nociception. Antagonizing hydrolysis by RGS4 in the vlPAG enhances morphine-mediated analgesia, but not fentanyl, which may be a function of their different signaling pathways (Morgan et al., 2020).

In contrast, RGS proteins positively modulate postsynaptic MOR-mediated GIRK activation in the vlPAG (McPherson et al., 2018). RGS proteins playing a facilitatory role in MOR-GIRK signaling is counterintuitive, as RGS proteins inactivate G proteins which activate GIRK channels. However, a “kinetic scaffolding” model outlines the necessity of rapid turnover of G proteins to replenish the inactive G protein substrate pool for quick re-activation by the receptor (Clark et al., 2003; Zhong et al., 2003). The proximity of substrates and binding partners, here MORs and GIRKs, allows for expedient activation→ channel gating → inactivation. As a result, when the RGS binding is disrupted in the RGS-insensitive mouse model, the efficiency in coupling is lost and the substrate pool turnover is hindered, reducing the overall K^+^ conductance through the GIRK channel. Thus, this model suggests that RGS proteins serve as key components in receptor and effector coupling, enhancing the efficiency of the signal transduction pathway. Distinct actions of RGS proteins and agonist-specific G protein recruitment, in pre- and postsynaptic MOR signaling provide another avenue for compartment-specific MOR signaling that can affect the analgesic circuit. Future studies on how acute and persistent pain states may influence RGS actions in pre- and postsynaptic MOR signaling will further our understanding of how RGS-mediated positive and negative modulation of compartment-specific MOR signaling within the vlPAG influence pain states. Furthermore, the duration of opioid (i.e., morphine) exposure impacts the association between MORs and specific RGS proteins in the PAG (Garzón et al., 2005a), highlighting one mechanism by which treating pain states with exogenous opioids can influence MOR regulation.

Sustained MOR activation can also produce heterologous desensitization at adjacent receptors that use the same intracellular signaling components (Leff et al., 2020; Adhikary et al., 2022). As a result, these mechanisms associated with MOR desensitization could be adapting the signaling of other receptors, which then influence tolerance and withdrawal. Additional receptors within the vlPAG have been hypothesized to contribute to the analgesic tolerance of opioids, such as the nociceptin receptor (NOP), which is another GPCR that is densely expressed within the vlPAG with similar homology to MOR/DOR/KOR but low affinity for opioid agonists and antagonists (Anton et al., 1996). Activation of NOP blocks analgesia and NOP antagonists microinjected into the vlPAG stop the development and expression of analgesic tolerance to systemic morphine administration (Parenti and Scoto, 2010; Scoto et al., 2010). Ongoing activation of NOP produces PKC-mediated heterologous desensitization of MORs in cultured cells (Mandyam et al., 2002), providing an example of how cross-talk between these receptor signaling systems can influence analgesic efficacy.

## Cellular Diversity

Many methods have been used to characterize different neuronal populations within brain regions. Tools that utilize genetic approaches to selectively alter neuronal activation, such as optogenetics or DREADDs, have reinforced our understanding of how the activation of excitatory and inhibitory vlPAG neurons influences the net output of the descending pain modulatory pathway. However, these studies do not address the question of which vlPAG neurons are recruited during acute and persistent pain states to influence ongoing nociception. Additional features such as intrinsic firing and membrane properties, receptor and channel expression, endogenous opioid peptide production, afferent inputs, and efferent targets can collectively define vlPAG neurons engaged by acute nociceptive stimuli, ongoing pain states, and endogenous or exogenous opioids. Other cell types within the vlPAG, such as microglia, also play important and extensive roles in the pain response and analgesia (Loyd and Murphy, 2006; Fullerton et al., 2018; Averitt et al., 2019).

### Neurotransmitter content

Early work using GAD-immunoreactivity, labeled a subset of ~33% of vlPAG cell bodies (Barbaresi and Manfrini, 1988; Reichling and Basbaum, 1990). The GABAergic subpopulation combined with the identification of a direct, excitatory connection between the vlPAG and the RVM that contributes to stimulation-mediated analgesia (Behbehani and Fields, 1979), leads to the proposed circuit where inhibitory neurons in the vlPAG serve as an interneuron population that control the intensity of the output signal to the RVM (Basbaum and Fields, 1984). To determine the effect of GABA on vlPAG output, GABA receptor antagonists were locally infused into the vlPAG increasing vlPAG firing and the firing of downstream RVM OFF-cells, producing analgesia (Moreau and Fields, 1986; Behbehani et al., 1990; Knight et al., 2002). These studies do not determine the source of GABA, which can come from GABAergic interneurons or GABAergic afferents originating from many different brain regions. Direct evidence for GABAergic interneurons within the vlPAG has not been provided to date.

Selective activation of vlPAG GABA neurons using DREADDs produces hyperalgesia and confirms the pronociceptive role of vlPAG GABA neurons independent of GABA afferents from other regions (Samineni et al., 2017a). However, this experiment does not rule out that this behavioral outcome could be the result of GABAergic neurons that project to the RVM that directly inhibit spontaneous OFF-cell firing (Heinricher et al., 1991). Histological studies confirm that GABAergic afferents in the RVM coming from the vlPAG come in contact with both OFF- and ON-cell populations (Morgan et al., 2008). Additionally, DREADD-mediated activation of GABAergic neurons does not address whether acute or ongoing nociceptive stimuli activate the same neurons within the vlPAG, demonstrating the physiological relevance of the impact of selectively activating this population or if neuronal activation is more heterogeneous, and if so, what resulting output that produces. Additionally, it is important to identify whether there are other circuit consequences of increased activity of vlPAG GABAergic neurons, such as increasing GABA release onto RVM OFF-cells, which would also produce hyperalgesia.

The proinflammatory cytokine Tumor Necrosis Factor-α (TNF-α) has been recently shown to selectively activate GABAergic neurons within the vlPAG (Pati and Kash, 2021), suggesting a possible mechanism by which a pain state can produce the targeted activation of vlPAG GABA neurons. TNF-α is one of many proinflammatory cytokines released by activated microglia, which are activated by inflammatory pain states (Fullerton et al., 2018). Interestingly, the enhanced activity of vlPAG GABA neurons by TNF-α did not increase GABAergic synaptic inputs onto neighboring vlPAG dopamine (DA) neurons—suggesting that if these GABAergic neurons send local collaterals within the vlPAG, they do not target DA neurons. Altogether, it is possible that pain states activate microglia, which release TNF-α, activating GABA neurons to enhance local GABA tone—resulting in descending facilitation through a specific subpopulation of vlPAG neurons. However, this possible mechanism would need to be confirmed in a pain model to implicate selective activation of vlPAG GABA neurons in altered pain modulation during pain states.

Selective activation of glutamatergic neurons in the vlPAG with DREADDs promotes analgesia (Samineni et al., 2017a). This reinforces the conclusion from many early studies that stimulation-mediated analgesia is driven by the activation of glutamatergic neurons (Reynolds, 1969; Mayer et al., 1971; Mayer and Liebeskind, 1974; Akil and Liebeskind, 1975; Behbehani and Fields, 1979; Soper and Melzack, 1982; Jensen and Yaksh, 1989). However, selective activation of glutamatergic vlPAG neurons also enhances anxiety (Taylor et al., 2019), one of the many off-target effects precluding this stimulation target as a therapeutic option for clinical pain management. Deep brain stimulation targeting the vlPAG has been applied therapeutically for treatment-resistant hypertension (Patel et al., 2011; O’Callaghan et al., 2014), emphasizing the many subcircuits that utilize this region and the importance of understanding whether specific stimuli engage different neuronal subpopulations within the vlPAG. Furthermore, a recent study using single nucleus RNA-sequencing and Multiplexed Error-Robust Fluorescence *in situ* Hybridization (MERFISH) identified over 100 excitatory and inhibitory neuronal populations (Vaughn et al., 2022). In addition to unique transcriptional profiles, these neurons were found to be spatially distributed uniquely along the rostral-caudal axis, and multiple populations were activated in unison by different instinctive behaviors (i.e., mating, aggression, etc.)—underscoring the complexity in subpopulations of excitatory and inhibitory vlPAG neurons.

An increasing number of studies are providing evidence that glutamate neurons do not represent a functionally homogeneous population of vlPAG neurons. One example is a Chx10-expressing subpopulation of glutamate neurons that are specifically involved in mediating freezing behaviors (Vaaga et al., 2020). Activation of another subpopulation vlPAG glutamate neurons that express dynorphin produces dynorphin-mediated facilitation of nociception through signaling at terminals in the RVM (Nguyen et al., 2022). Findings such as these emphasize the caution we should take when using neurotransmitter content as the only genetic marker in behavioral studies. Although it is interesting to know that the net output of activating all glutamate neurons is analgesia, important questions remain. First, how many vlPAG glutamate neurons are necessary to produce analgesia? Second, do glutamate neurons that promote descending inhibition overlap with glutamate populations involved in other outputs (i.e., freezing)? Lastly, are there markers for subpopulations of glutamate neurons that are activated by nociceptive stimuli or necessary for producing analgesia that can be harnessed to develop targeted drug delivery methods? Together, the answers to these questions will equip us with the information needed to develop therapeutic manipulations that produce the smallest intervention possible that drives descending inhibition from the level of the vlPAG.

Additional neuron populations within the vlPAG with different neurotransmitter content engage with descending modulation differently. Most notably, DA neurons have been implicated in the broader supraspinal pain circuitry and analgesia (Hökfelt et al., 1976; Meyer et al., 2009; Taylor et al., 2019; Yu et al., 2021) despite not projecting directly to the RVM (Suckow et al., 2013). Interestingly, these DA neurons co-release both DA and glutamate at terminals in the bed nucleus of the stria terminalis (BNST; Li et al., 2016). Selective activation of these vlPAG DA neurons produces antinociception in male rats (Yu et al., 2021). Serotonergic neurons that are densely populated in the dorsal raphe and extend diffusely up into the most ventral portion of the vlPAG (Crawford et al., 2010), have also been implicated in opioid-mediated analgesia (Samanin et al., 1970).

Altering the activity of vlPAG neuron populations with distinct neurotransmitter content can influence pain modulation, however, this alone does not answer important questions: (1) are these molecularly defined subpopulations selectively engaged by pain states, mirroring these activation/inhibition studies in a physiological condition; (2) how does this change over the course of acute, persistent, and chronic stages; and (3) how do endogenous and exogenous opioids influence how these neurons participate in descending circuitry in naïve and pain states?

### Receptor or channel expression

In addition to neurotransmitter content, the expression of receptors and channels amongst vlPAG neurons can differentiate distinct populations and potentially define any selective, population-specific engagement by pain states or opioids (Chieng and Christie, 1994a; Park et al., 2010; Liao et al., 2011; Du et al., 2013; Lau and Vaughan, 2014; McDermott et al., 2019). Although MORs mediate morphine antinociception (Matthes et al., 1996) all three opioid receptors (MOR, DOR and KOR) are expressed in vlPAG. DOR and KOR are densely expressed within the vlPAG, both on cell bodies (including RVM-projecting neurons) and on afferent terminals (Mansour et al., 1986; Kalyuzhny et al., 1996; Gutstein et al., 1998; Kalyuzhny and Wessendorf, 1998; Wang and Wessendorf, 2002). Neither DOR nor KOR activation elicits GIRK currents from vlPAG neurons in rats (Chieng and Christie, 1994a), although both activate GIRK currents in mouse vlPAG (Vaughan et al., 2003). Interestingly, DOR activation alone does not produce analgesia but potentiates MOR-mediated analgesia (Rossi et al., 1994). This effect is observed when MOR agonist DAMGO is microinjected into the vlPAG or RVM and DOR agonist deltorphin is microinjected into the other region and not when they are microinjected into the same region—suggesting synergy is occurring at the circuit and not cellular level.

Several studies have tried to use MOR as a marker for a specific functional subpopulation of vlPAG neurons. One interesting possibility identified in mice showed that MOR expressing, tonic firing GABAergic neurons also expressed T-type calcium channel, indicated by low-threshold spiking (LTS; Park et al., 2010). Using MOR-mediated GIRK currents, they observed that T-type channel expressing GABAergic neurons were opioid-sensitive (five neurons) and the remaining GABAergic (four neurons) and phasic firing, non-GABAergic neurons were opioid-insensitive. However, in a larger data set in the vlPAG of rats, LTS was not a predictor of opioid sensitivity (McPherson et al., 2021). Furthermore, LTS was observed in phasic firing neuronal populations in rats in addition to the tonic firing populations that exclusively had LTS in the mouse study. Together these discrepancies suggest either T-type channels are more broadly expressed in rats than in mice or the mouse data set did not capture a large enough sample to observe phasic firing, non-GABAergic, opioid-sensitive neurons.

Other receptors expressed in the vlPAG can also modulate the effects of opioids. For example, activating NOP with the endogenous ligand nociception/orphanin FQ reduces the analgesic efficacy of endogenous opioids and systemic morphine (Mogil et al., 1996). Conversely, NOP antagonists potentiate DAMGO efficacy, whether the animal is pretreated or given the antagonist after DAMGO administration (all microinjected within the vlPAG; Scoto et al., 2007). NOP activation also appears to be contributing to the development and expression of allodynia in acute inflammatory pain and chronic neuropathic pain (Scoto et al., 2009), making it a potential therapeutic target to both modulate opioid efficacy and nociceptive thresholds in the absence of opioid use.

Additionally, recent studies found that GPR171, a recently deorphanized GPCR, is expressed on GABAergic vlPAG neurons where it regulates opioid-mediated antinociception (McDermott et al., 2019). GPR171 agonists enhance morphine efficacy while antagonists do the opposite, with the most substantial effect seen with the supraspinal antinociceptive test (hotplate). The GPR171 agonist MS15203 administered daily after injury alleviated thermal hypersensitivity (after CFA) and allodynia (after neuropathic pain) in males only (Ram et al., 2021). Of note, the neuropathic pain model reduced PAG GPR171 expression in male mice only, which was recovered by the agonist treatment.

The DA and opioid receptor systems provide examples of signaling interactions that influence pain modulation at the level of the vlPAG. Activation of vlPAG DA receptors directly with agonist (-) apomorphine or indirectly with D-amphetamine, produces robust antinociception *via* the descending circuit with the RVM, and is attenuated by D_2_ receptor blockade (Flores et al., 2006; Meyer et al., 2009; Ferrari et al., 2021). In addition to DA-mediated antinociception, blocking either D_1_ or D_2_ DA receptors inhibits opioid-mediated antinociception in a dose-dependent manner (Flores et al., 2004; Meyer et al., 2009; Tobaldini et al., 2018). These results are consistent with previous findings that show a significant reduction in the antinociceptive effect of systemic opioids (specifically, heroin and morphine) after selectively ablating DA neurons within the vlPAG (Flores et al., 2004). Mechanistically, activation of D_2_ receptors induces GIRK currents (Pillai et al., 1998; Marcott et al., 2014) and dopamine applied on slices *in vitro* reduces presynaptic GABA release (Meyer et al., 2009). Interestingly, unlike the antinociceptive tolerance observed with repeated opioid administration, the DA-receptor system sensitizes to repeat (-) apomorphine administration, producing increased antinociception, making the furthered understanding of these mechanisms of particular relevance for the development of novel therapeutics (Schoo et al., 2018).

Overall, defining the specific combinations of receptor and channel expression in combination with other features of cellular heterogeneity (neurotransmitter content, intrinsic properties, and specific circuitry) will increase our understanding of neuron types within the vlPAG. Compiling these features into comprehensive vlPAG neuron profiles may provide interesting insight into how pain states alter these neurons, the descending modulatory circuit, and the efficacy of drugs targeting these receptor-channel complexes.

### Intrinsic firing properties

Characterizing intrinsic membrane and firing properties is a common approach to defining neuronal heterogeneity (Prescott and De Koninck, 2002; Sedlacek et al., 2007; Van Aerde and Feldmeyer, 2015; Pradier et al., 2019) and determining these properties in naïve animals allows for the evaluation of alterations induced by persistent inflammation (Li and Sheets, 2018; Adke et al., 2021; McPherson et al., 2021). Neuronal firing properties and response to noxious stimuli have been used to define important, functionally distinct neurons within the RVM (Fields et al., 1983a; Vanegas et al., 1984b). These landmark papers that characterized responses of distinct neuron types to noxious stimuli (ON-, OFF-, and NEUTRAL-cells) have served as a useful framework for subsequent findings.

ON- and OFF-cells respond differently to opioids, application of EAAs, and blocking inhibitory inputs. First, RVM ON-cells selectively express MORs and as a result, iontophoretic application of morphine inhibits ON-cell firing without affecting OFF-cell firing (Heinricher et al., 1992). ON- and OFF-cells respond differently to excitatory and inhibitory afferent input. Iontophoretic application of a glutamate receptor antagonist reduces the ON-cell burst triggered by the noxious stimulus and ON-cell spontaneous firing and does not alter OFF-cell firing (Heinricher and Roychowdhury, 1997; Heinricher et al., 1999). Conversely, iontophoretic application of the GABA antagonist bicuculline eliminates the OFF-cell pause triggered by the noxious stimulus but does not change ON-cell firing (Heinricher et al., 1991). Together these studies suggest that enhanced glutamate release within the RVM can increase ON-cell firing while keeping the OFF-cells unaltered and enhanced GABA release within the RVM can reduce OFF-cell firing without impacting ON-cell firing. This highlights the importance of identifying which vlPAG neurons are activated by pain states, how they alter afferent inputs in the RVM, and where endogenous or exogenous opioids intervene in the circuit.

In addition to determining how specific synaptic inputs can affect RVM ON- and OFF-cells, studies have examined how these cells respond to noxious stimuli during different pain stages. Upon CFA injection, both ON- and OFF-cell spontaneous activity are enhanced but spontaneous firing for both neuron types returns to baseline after a couple of hours; however, mechanical thresholds are reduced into the innocuous range (Cleary et al., 2008). Furthermore, blocking excitatory afferent inputs within the RVM prior to chronic constriction injury results in slower and diminished development of mechanical allodynia, correlating with a reduction in the hyperexcitability of spinal neurons (Sanoja et al., 2008). Combined with what is known about the effect of afferent inputs that impinge onto distinct neuron types in the naïve condition, this suggests that glutamatergic inputs onto ON-cells are important for the development of hyperalgesia, calling into question how excitatory and inhibitory projections from the vlPAG contribute to these changes.

*In vivo* recordings from the vlPAG have also identified neurons that respond to nociceptive stimuli (Heinricher et al., 1987; Samineni et al., 2017b), finding ON-, OFF-, and NEUTRAL-cells. Neuropathic pain induced by paclitaxel, a commonly used chemotherapy drug, enhances spontaneous firing and lowers the response thresholds in vlPAG ON-cells and OFF- and NEUTRAL-cells in response to noxious and previously innocuous stimuli (Samineni et al., 2017b). These studies provided evidence that pain states selectively activate subpopulations of neurons within the vlPAG and that the acute firing response to a noxious stimulus can be used to distinguish distinct vlPAG neuron populations.

An *ex vivo* survey of nearly 400 neurons using *in vitro* whole-cell patch-clamp experiments identified four distinct neuron types based on their intrinsic firing properties: Tonic (35%), Phasic (46%), Onset (10%), and Random (9%; McPherson et al., 2021). Tonic neurons (35%) fired continuously in response to depolarizing current steps compared to Phasic neurons (46%) which reached depolarization block in the more strongly depolarizing steps. These neuron types allowed the same study to identify that persistent CFA-induced inflammation (5–7 d) selectively enhances the spontaneous firing rate of Phasic neurons. Identifying activation of specific subtypes of vlPAG neurons prompts many interesting follow-up studies, including examining intrinsic changes in receptors and/or channels or adaptations in afferent inputs. A study evaluating GABAergic neurons in a genetically defined mouse model observed that firing patterns in mice largely correlated with neurotransmitter content, with 31/33 GABAergic neurons having a tonic firing pattern with the other 2/33 showing a phasic pattern (Park et al., 2010). If this correlation observed in mice is upheld in rats, enhanced spontaneous activity of Phasic neurons after persistent inflammation may be producing the glutamate afferent input onto RVM ON-cells that contributes to allodynia. These interpretations are made even more interesting if neurons with distinct firing patterns have unique afferent inputs, that could for example contribute to enhanced Phasic firing after persistent inflammation or unique efferent targets that implicate the enhanced Phasic firing in altering signaling within different circuits.

In addition to providing a useful framework to identify mechanisms of targeted neuronal activation after different stimuli, firing patterns provide insight into how neurons may encode noxious stimuli. For example, a tonic firing neuron can entrain stimuli of varying intensities, whereas a phasic neuron can only do so at low-intensity ranges. At the higher depolarizing intensities a Phasic neuron becomes a coincidence detector, similar to the Onset neuron (Prescott and De Koninck, 2002). This can change whether presynaptic release from these neurons onto their downstream targets is ongoing (Tonic) or transient (Phasic). Recently published work has discovered opposing functional outputs produced by activating the same GABAergic neuron population with different channelrhodopsin-2 variants that have distinct off-kinetics (Baleisyte et al., 2022). The two variants produce two different firing patterns with the same optogenetic stimulation paradigm; the faster variant has identical action potentials with each stimulation, whereas the slower variant leads to significant attenuation of the action potential peak over repeated stimulation—demonstrating the importance of combining firing properties with neurotransmitter content to more completely understand the implication of neuron populations within a circuit.

## Circuit Diversity

### Afferent inputs

The vlPAG receives inputs from many cortical and subcortical regions associated with nociceptive, cognitive, and affective components of pain. Ascending nociceptive inputs to the vlPAG come through the spinothalamic, spinoparabrachial, and spinomesocenphalic tracts, with some inputs coming directly from the spinal cord to the vlPAG (Menétrey et al., 1982; Yezierski and Mendez, 1991). The spinomesencephalic tract provides direct inputs to the PAG, however, these inputs have been linked to nociception and analgesia, as well as aversive behaviors (Willis and Westlund, 1997). Additional ascending nociceptive inputs come from the parabrachial complex (Gauriau and Bernard, 2002), which receives inputs from the superficial and deep dorsal horn (Roeder et al., 2016). Forebrain regions including the medial prefrontal, agranular insular, and anterior cingulate cortices, amygdala, BNST, and hypothalamus send the most significant supraspinal inputs to the PAG (Shipley et al., 1991; An et al., 1998; Floyd et al., 2000; Hao et al., 2019; Silva and McNaughton, 2019). In addition to anatomical studies showing connections between these regions and the vlPAG, studies using lesions and pharmacological manipulations have provided evidence that these regions participate in pain circuitry (Donahue et al., 2001; Ikeda et al., 2007; Starr et al., 2009; Bliss et al., 2016; Mills et al., 2018). For example, antinociception induced by morphine injected into the basolateral and medial nuclei of the amygdala is interrupted by lesioning the vlPAG (Helmstetter et al., 1998; McGaraughty et al., 2004)—emphasizing the importance of the vlPAG as an integration site for cortical inputs involved in pain modulation and opioid-mediated analgesia.

Supraspinal inputs are both excitatory and inhibitory so vlPAG neuronal activity is dictated by the E/I balance onto an individual neuron. Opioid-mediated disinhibition of pain is one example where it is presumed that glutamatergic PAG output neurons are biased towards a more inhibited state by GABAergic afferent inputs. In one study, glutamatergic inputs from the medial (fastigial) cerebellar nuclei synapse onto 20% of Chx10-expressing glutamatergic neurons, 21% of GABAergic (GAD2^+^) neurons, and 70% of DA neurons within the vlPAG (Vaaga et al., 2020), clearly demonstrating that afferent inputs are not universally distributed within the vlPAG. These results highlight the importance of identifying specific afferent inputs that are activated by either pain or opioids that could be useful in defining subpopulations of vlPAG neurons.

Persistent inflammation induced with CFA enhances GABA tone in the vlPAG of female rats (Tonsfeldt et al., 2016). In addition to changes in GABA release by pain states, the glutamatergic release was decreased in the vlPAG 3 and 10 d after spinal nerve ligation (Ho et al., 2013). Although different pain models were used in these studies, the results suggest possible changes in the balance of excitatory and inhibitory inputs (E/I balance). Altered afferent release from either excitatory or inhibitory terminals can influence firing rates, and the changes to firing induced by opioid-mediated inhibition of presynaptic release, thus yielding altered engagement with downstream targets like the RVM. For example, if the afferent inputs onto a neuron are excitatory-dominant in the naïve condition, opioids will remove the excitatory drive, resulting in inhibition of firing. However, if the known enhanced GABA tone after persistent pain shifts the E/I balance onto this same neuron to becoming inhibitory-dominant, opioids will now activate firing. This shift in how opioids can alter vlPAG neuronal firing can have a significant effect when considering how any specific neuron engages with ON- or OFF-cells within the RVM.

### Efferent targets

Projection target is another important feature that can increase our understanding of how distinct types of vlPAG neurons engage with downstream targets and how that connection is altered by persistent inflammation or opioid action. The vlPAG contributes to the overall output of the descending pain modulatory pathway at the level of the dorsal horn of the spinal cord through its connection with the RVM (Behbehani and Fields, 1979; Gebhart et al., 1983; Prieto et al., 1983). The RVM-projecting population contains both GABAergic and non-GABAergic neurons (Commons et al., 2000; Morgan et al., 2008). In the mouse, both tonic firing (7/12) and phasic firing (5/12) neurons project to the RVM with comparable density; however, low-threshold spiking, MOR-expressing GABAergic tonic firing neurons did not project to the RVM (Park et al., 2010). Lau et al. (2020) found that RVM-projecting vlPAG neurons lacking MOR expression are disinhibited by DAMGO application compared to non-RVM-projecting neurons which are inhibited (*n* = 9), however, other findings show that RVM-projecting vlPAG neurons can express MORs (Commons et al., 2000). A subset of dynorphin-releasing glutamatergic vlPAG neurons (~32%) project to the RVM, making up ~10% of the RVM-projecting vlPAG neurons (Nguyen et al., 2022). Altering the activity of this particular subpopulation of excitatory neurons can impact responses to cold, thermal, itch, and nociception.

Recent studies have also shown that vlPAG projections to regions other than the RVM can be implicated in antinociception, expanding the definition of subpopulations involved in descending pain modulation beyond RVM-projecting neurons. One example is DA neurons that project to the BNST (Hasue and Shammah-Lagnado, 2002; Yu et al., 2021), which interestingly has reciprocal connections with the vlPAG via GABAergic efferents (Hao et al., 2019). Despite not projecting to the RVM (Suckow et al., 2013), these DA neurons have been implicated in the broader supraspinal pain circuitry and analgesia (Meyer et al., 2009; Taylor et al., 2019; Yu et al., 2021). Another is the connection between the central medial nucleus of the thalamus, which when lesioned temporarily alleviates mechanical hyperalgesia in a neuropathic pain model (Sun et al., 2020). Additional examples of reciprocal connections between the vlPAG and other brain regions, such as the amygdala (Ottersen, 1981; Hasue and Shammah-Lagnado, 2002; Oka et al., 2008; Sun et al., 2019), show pain-induced alterations (Li and Sheets, 2018). Multi-region circuits, such as that between the vlPAG, central medial thalamic nucleus, and the BLA are activated by neuropathic pain (Sun et al., 2020), which is known to project back to the vlPAG via neurons with distinct intrinsic membrane properties within the central medial and lateral nuclei of the amygdala (Rizvi et al., 1991; Li and Sheets, 2018). These reciprocal connections could account for the polysynaptic responses that lead to latent changes in RVM neuronal firing in response to vlPAG stimulation (Odeh et al., 2003).

The vlPAG has many other efferent targets that are associated with other behaviors. GABAergic projections to the VTA have been implicated in freezing behaviors (Laurent et al., 2020). Single-unit recordings in awake behaving animals have linked vlPAG cellular activity to threat probability evaluation (Wright et al., 2019). The subpopulation of neurons in mice involved in freezing with distinct connectivity, molecular markers (Chx10 and glutamate), and electrophysiological features (Vaaga et al., 2020). An entire field of work has implicated this region in the acquisition, expression, and extinction of fear, anxiety, or defensive response (Borszcz et al., 1989; Fanselow, 1991; De Oca et al., 1998; McDannald, 2010; Wright and McDannald, 2019; Wright et al., 2019). It is important to understand whether the circuits associated with behaviors or physiological states other than pain overlap with the vlPAG neurons that are specifically engaged in pain modulation. This could shed light on possible circuit mechanisms for comorbidities observed with chronic pain or other conditions that increase an individual’s susceptibility to developing pain conditions.

## Conclusion

The heterogeneity of the vlPAG calls for understanding neuronal subpopulations that comprise pain circuits with a greater resolution than the field currently uses. Combining multiple features, such as neurotransmitter content, receptor/channel expression, intrinsic firing properties, afferents inputs, efferent targets, etc., will create the opportunity to identify novel targets that interfere with pain processing, especially in chronic pain states. As new innovative approaches are developed, we can address key questions that remain in the field regarding the spatial and temporal specificity of endogenous opioid release within the descending pain modulatory pathway.

## Author Contributions

KM and SI wrote the article. All authors contributed to the article and approved the submitted version.

## Funding

This work was supported by the NIH NIDA DA042565 (SI), the National Science Foundation Graduate Research Fellowships Program, and the Lacroute Fellows Program (The Vollum/OHSU-NGP).

## Conflict of Interest

The authors declare that the research was conducted in the absence of any commercial or financial relationships that could be construed as a potential conflict of interest.

## Publisher’s Note

All claims expressed in this article are solely those of the authors and do not necessarily represent those of their affiliated organizations, or those of the publisher, the editors and the reviewers. Any product that may be evaluated in this article, or claim that may be made by its manufacturer, is not guaranteed or endorsed by the publisher.
